# An Experience Oriented-Convergence Improved Gravitational Search Algorithm for Minimum Variance Distortionless Response Beamforming Optimum

**DOI:** 10.1371/journal.pone.0156749

**Published:** 2016-07-11

**Authors:** Soodabeh Darzi, Sieh Kiong Tiong, Mohammad Tariqul Islam, Hassan Rezai Soleymanpour, Salehin Kibria

**Affiliations:** 1 Center of System and Machine Intelligence, College of Engineering, Universiti Tenaga Nasional, Selangor, Malaysia; 2 Power Engineering Center, Universiti Tenaga Nasional, Selangor, Malaysia; 3 Department of Electrical, Electronic & Systems Engineering, Universiti Kebangsaan Malaysia, Selangor, Malaysia; 4 Department of Electrical Engineering, Semnan University, Semnan, Iran; Beihang University, CHINA

## Abstract

An experience oriented-convergence improved gravitational search algorithm (ECGSA) based on two new modifications, searching through the best experiments and using of a dynamic gravitational damping coefficient (*α*), is introduced in this paper. ECGSA saves its best fitness function evaluations and uses those as the agents’ positions in searching process. In this way, the optimal found trajectories are retained and the search starts from these trajectories, which allow the algorithm to avoid the local optimums. Also, the agents can move faster in search space to obtain better exploration during the first stage of the searching process and they can converge rapidly to the optimal solution at the final stage of the search process by means of the proposed dynamic gravitational damping coefficient. The performance of ECGSA has been evaluated by applying it to eight standard benchmark functions along with six complicated composite test functions. It is also applied to adaptive beamforming problem as a practical issue to improve the weight vectors computed by minimum variance distortionless response (MVDR) beamforming technique. The results of implementation of the proposed algorithm are compared with some well-known heuristic methods and verified the proposed method in both reaching to optimal solutions and robustness.

## 1.Introduction

Adaptive array techniques have been utilized to mitigate multipath fading and high co-channel interference in mobile, wireless, radar communications, satellite and many other similar applications to achieve robust performance and high data transmission rate [1]. One of the known array processing techniques is the minimum variance distortionless response (MVDR) technique, or Capon’s algorithm [2]. This approach is typically applied to cancel unwanted signals (interfering signals) and generate a strong beam towards the desired signal through its computed weight vectors. However, MVDR may have unacceptably low nulling in dynamic and various interference scenarios [3]. Recently, enormous amount of research has been dedicated to applying several optimization approaches for such problems. Thus, approaches such as genetic algorithms (GA) [4, 5], tabu search (TS) [6–8], particle swarm optimization (PSO) [9, 10], ant colony optimization (ACO) [11–13], differential evolution (DE) [14], clonal selection (CS) [15, 16], artificial bee colony (ABC) [17] and artificial immune system (AIS) [18,19] have been suggested and implemented to improve the robust performance, like interference cancelling, in antenna systems.

Recently, GSA was presented as a heuristic optimization algorithm inspired by Newtonian laws of gravitation. It was shown to outperform similar algorithms like PSO and GA for common benchmark functions [20]. Since the inception of GSA, a large number of researchers have introduced various modified and improved gravitational search algorithms based on the idea of memory and social information of PSO, novel strategies to define the agents’ search pattern along with other stochastic strategies [21–26]. The effectiveness of GSA and its version for binary encoded problems (BGSA) [27] in solving a set of nonlinear benchmark functions has been proven. However, very few of these GSA variants have been implemented for the beamforming applications [28].

In this study, we propose an experience oriented-convergence improved gravitational search algorithm (ECGSA) to deal with complicated optimization problems like the MVDR. ECGSA benefits from two modifications: one is to save the best fitness function evaluation of the agents during search process and to treat them as the agents’ effective positions in terms of applying force to other agents. By this modification, no discovered optimal trajectories are lost during the search process and also the agents can search through these optimal trajectories while avoiding local optimums. This is contrary to GSA, in which, the search trajectory is unstable as the agents move stochastically without any feedback to their best experiments. In ECGSA, if any agent finds better solution it updates its best position to its current location. The second modification is to use a dynamic gravitational damping coefficient parameter known as *α*. The proposed dynamic *α* is introduced to balance the exploration and exploitation properties of the standard GSA. With a relatively low value of *α* during first stage of search process, the agents can have larger velocities for a better exploration. On the other hand, the agents can converge to the optimal solution effectively at the final stage of the search process by rapidly increasing the value of *α*, consequently, reducing the agents velocities.

The most significant difference between stochastic leader gravitational search algorithm (SLGSA) [28] and ECGSA is that ECGSA retains memory of the results from previous iterations, whereas, SLGSA is memory less (i.e. uses results from current iteration only). This results in loss of optimal trajectory in SLGSA. Furthermore, SLGSA uses a stochastic method to determine which agents are allowed to influence other agents. This increases the exploration process by increasing diversity, hence, preventing premature convergence. ECGSA uses the more conventional approach to maintain diversity in the early phase by using high value of gravitational coefficient function, *G(t)*. Hence, ECGSA is fundamentally less stochastic than SLGSA. Thus ECGSA converges with greater precision than SLGSA. As SLGSA has the stochastic method of increasing diversity, it does not use the activation ratio, *σ* parameter. Overall, the two algorithms provide fundamentally different approaches to balancing the exploration and exploitation aspects of the search process.

In this study, the ECGSA based MVDR has been used in beamforming with uniform linear antenna arrays of 0.5λ spacing between adjacent elements and radiating at 2.3 GHz. The proposed ECGSA is also verified by testing it on some well-known complicated optimization problems. The rest of the paper is organized in the following order: Section 2 presents a brief review of GSA. Our proposed ECGSA is introduced in section 3. The validation of the proposed ECGSA via various functions and the simulation results are reported in section 4. Section 5 presents the basics of the conventional MVDR. Section 6 demonstrates the integration of MVDR with ECGSA along with performance comparison of conventional MVDR with MVDR-PSO, MVDR-GSA and MVDR-ECGSA in different interference scenarios. Finally, Section 7 presents concluding remarks about this investigation.

## 2.Gravitational Search Algorithm

**Fig 1:**
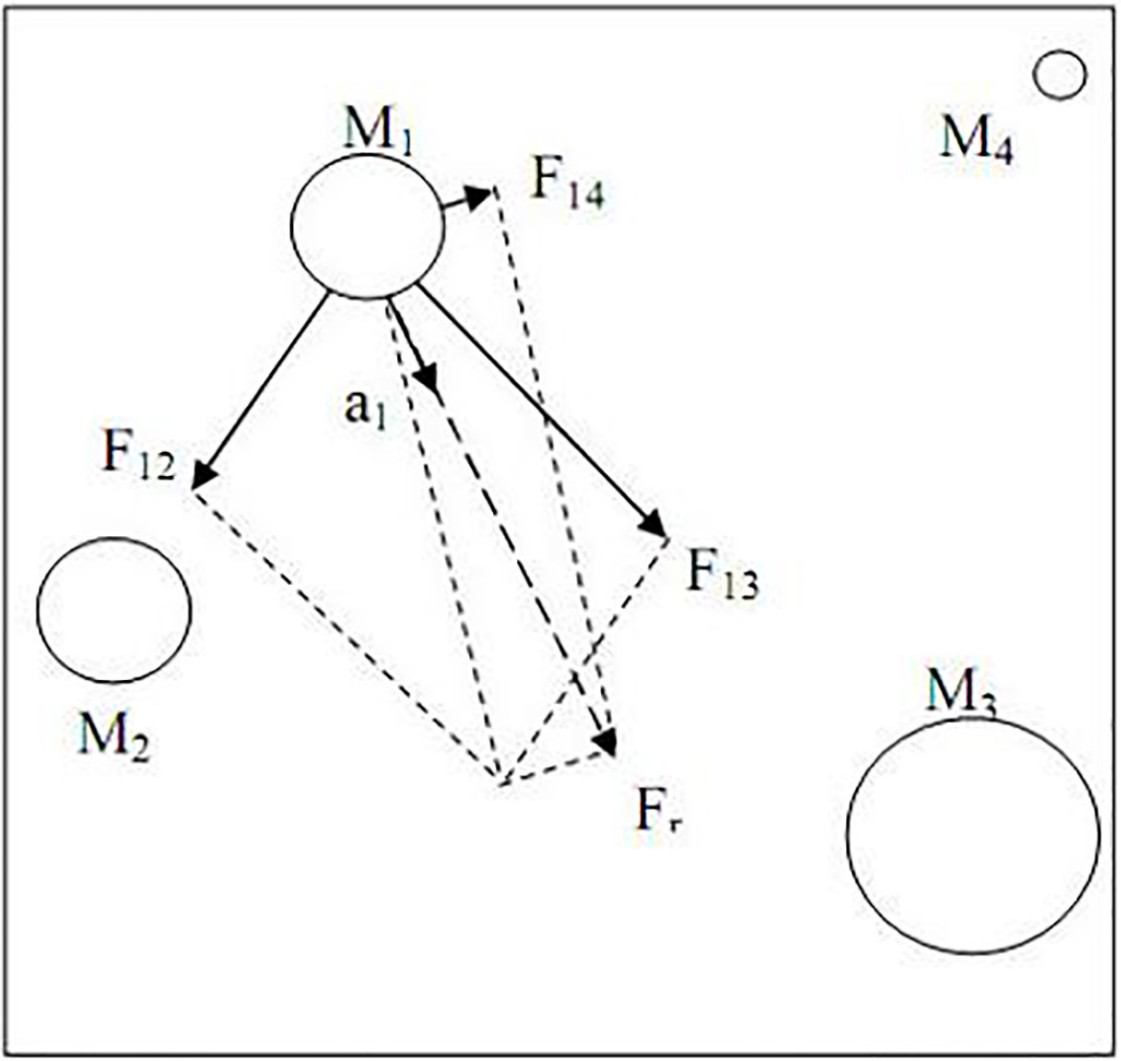
Mass acceleration toward the result force in GSA [20].

The GSA algorithm was proposed by Rashedi, et al. [20, 28] as a global optimization method in 2009. The GSA agent’s movements are estimated through their masses. All the GSA search agents gravitate toward the agents with heavier masses due to their gravitational attraction as shown in Fig 1. Agents (masses) with better fitness value in GSA will correspond to larger masses.

For the *i*th agent of *N* agents of GSA, the position vector is defined by:

$$X_{i}=\left(x_{i}^{1},\ldots ,x_{i}^{d},\ldots ,x_{i}^{n}\right),\;i=1,2,\ldots ,N$$ (1)

where *x*_*i*_^*d*^ represents the position of the *i*th agent in the *d*th dimension (decision variable), and *n* represents the total number of decision variables. Inertia masses are calculated by the fitness evaluation as follows:

$$m_{i}(t)=\frac{fit_{i}(t)-worst(t)}{best(t)-worst(t)}$$ (2)

$$M_{i}(t)=\frac{m_{i}(t)}{\sum_{j=1}^{N}m_{j}(t)}$$ (3)

where *fit*_*i*_*(t)* represents the fitness value of the *i*th agent at iteration *t*; *worst(t)* and *best(t)* represent the worst and best fitness of all agents, respectively; and *M*_*i*_*(t)* represents the mass value of the *i*th agent at iteration *t*. The force operating on mass i from mass j can be calculated as follows:

$$F_{ij}{}^{d}(t)=G(t)\frac{M_{i}(t)\times M_{j}(t)}{R_{i,j}(t)+\varepsilon }\left(x_{j}{}^{d}(t)-x_{i}{}^{d}(t)\right)$$ (4)

where *M*_*i*_ and *M*_*j*_ represent masses of the *i*th and *j*th agents respectively; *ε* is a significantly small constant that prevents division by zero; *R*_*i*,*j*_*(t)* represents the Euclidean distance between the *i*th and *j*th agents defined as follows:

$$R_{i,j}(t)={\left\|X_{i}(t),X_{j}(t)\right\|}_{2}$$ (5)

and *G(t)* is the gravitational coefficient at iteration *t*. The gravitational coefficient *G(t)* is a monotonically decreasing function of time when it is set to *G*_*0*_ at the starting and will be exponentially damped to examine the accuracy of the algorithm according to the equation below:

G(t)=G0×exp(−α×ttmax) (6)

where *α* is gravitational damping coefficient which is constant in GSA, *t* is the current iteration and *t*_*max*_ is the maximum iteration number. The total force affecting the *i*th agent in the *d*th dimension is calculated as follows:

$$F_{i}^{d}(t)=\sum_{j\in k\;best,\;j\neq i}rand_{j}F_{ij}^{d}(t)$$ (7)

where *kbest* is a time dependent function that is able to qualify the exploitation and exploration. It is initialized to the total number of agents (*k*_*0*_) at the start and linearly decreased to one with time [20]. *rand*_*j*_ is a random number between zero and unity. By the laws of motion, $a_{i}^{d}$, the acceleration of agent *i* in dimension *d*, could be obtained as follows:

$$a_{i}^{d}(t)=\frac{F_{i}^{d}(t)}{M_{i}(t)}=\sum_{j\in k\;best,j\neq i}rand_{j}G(t)\frac{M_{j}(t)}{R_{i,j}(t)+\varepsilon }\left(x_{j}^{\;d}(t)-x_{i}^{\;d}(t)\right)$$ (8)

The position of agent *i* depends on the agent velocity and it can be updated using the following equations:

$$v_{i}^{\;d}(t+1)={rand}_{i}\times v_{i}^{\;d}(t)+a_{i}^{\;d}(t)$$ (9)

$$x_{i}^{\;d}(t+1)=x_{i}^{\;d}(t)+v_{i}^{\;d}(t+1)$$ (10)

where *v*_*i*_^*d*^*(t)* represents the velocity of agent *i* in dimension d at iteration *t*; and *rand*_*i*_ is a random number between zero and unity.

## 3.Experience Oriented-Convergence Improved Gravitational Search Algorithm

In this study, two distinct modifications, using the best found experiments as the agents’ present positions and introducing a dynamic gravitational damping coefficient value, are introduced to GSA as follows:

### A. Using the best found experiments of agents as their present positions

The standard GSA does not retain the best fitness function evaluations that the agents find during search process. This is in contradiction of other stochastic search algorithms such as PSO [29], GA [30], DE [31] and simulated annealing (SA) [32]. GSA only relies on the current information of the agents to determine the present search trajectory. This is a weakness of GSA, because the optimal found trajectories may be lost due to updating them by non-optimal new ones. In this paper, we overcome this defect by using the best fitness function evaluations of the agents as their present positions for calculating new movements. To do this, the position updating (10) is modified as follows:

$$x\;{}_{i}^{d}(t+1)=\left\{ \begin{array}{l} {\bar{x}}_{\;i}^{d}\;\text{if}\;{\bar{X}}_{i}\;\text{is better than}\;X_{\;i}(t)\\ x\;{}_{i}^{d}(t)\;\text{otherwise} \end{array}\right.$$ (11)

Where ${\bar{x}}_{i}^{\;d}$ represents the unconfirmed position of agent *i* in dimension *d* which is calculated from the following equation:

$${\bar{x}}_{\;i}^{d}=x\;{}_{i}^{d}(t)+v\;{}_{i}^{d}(t+1)$$ (12)

And ${\bar{X}}_{i}$ is the unconfirmed position vector of agent *i* containing ${\bar{x}}_{i}^{\;d}$.

Eq (11) simply states that the new movement of an agent is accepted to be its new position, if the movement results in a better solution, otherwise the position of the agent remains unchanged at the present iteration. By this modification no optimal found trajectory is lost, and also the movement of any agent is started from its best experiment at any iteration, which achieves good local search around the optimal found solutions.

### B. Dynamic gravitational damping coefficient value

**Fig 2:**
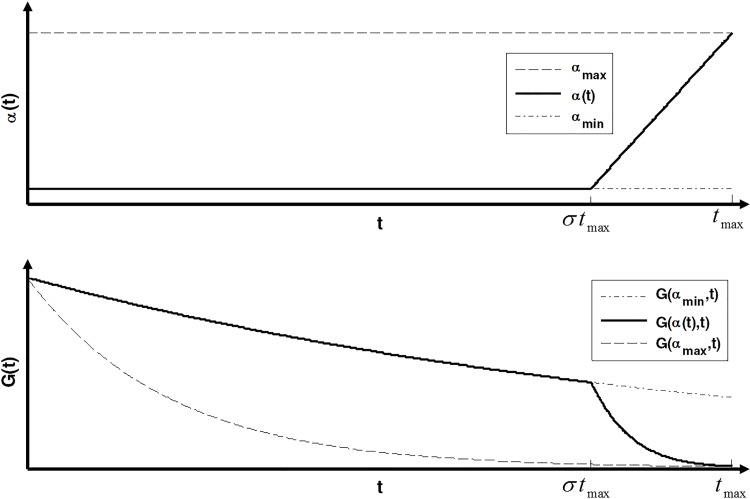
The variations of the proposed *α(t)* along with *G(t)*.

Ideally, a stochastic search based algorithm should focus on exploration and follow it with exploitation. The exploration phase allows the algorithm to gather information about the overall search space. Once the search space is sufficiently explored, the focus can be shifted to exploiting the information gathered by exploration. In GSA, the gravitational coefficient *G(t)* balances the exploration and exploitation capabilities through increasing/decreasing the magnitude of agent acceleration vector. Higher values of *G(t)* are needed for relatively large movements at initial stages of search process, while lower values are required for a better convergence to optimal solution at the final phase. The value of *G(t)* is exponentially decreased during iterations according to (6). The gravitational damping coefficient *α* adjusts the degree of damping. The more gravitational damping coefficient *α* is, faster the gravitational coefficient *G(t)* drops. But finding a value of *α* to be optimal for both exploration and exploitation is a hard decision. If *α* is too high, the algorithm suffers from premature convergence. Similarly, if it is too low, the algorithm couldn’t converge to the optimal region at the final iterations. In ECGSA, we propose a gravitational damping coefficient *α* according to the following equation:

$$\alpha (t)=\left\{ \begin{array}{l} \alpha_{\text{min}}\;,\;\text{if}\;\frac{t}{t_{\text{max}}}<\sigma \\ \alpha_{\text{min}}+\frac{\left(\alpha_{\text{max}}-\alpha_{\text{min}}\;\right)}{\left(1-\sigma \right)}(\frac{t}{t_{\text{max}}}-\sigma ),\;\text{otherwise} \end{array}\right.$$ (13)

where *σ* is a constant value between 0 and 1, representing the ratio of the first sub function being activated. The variations of the proposed *α(t)* along with *G(t)* are shown on Fig 2.

According to Fig 2, the value of *α* is constant and relatively low for a large portion of iterations resulting in good exploration capability. It is linearly increased to a high value at the final iterations resulting in good exploitation and convergence behavior.

**Fig 3:**
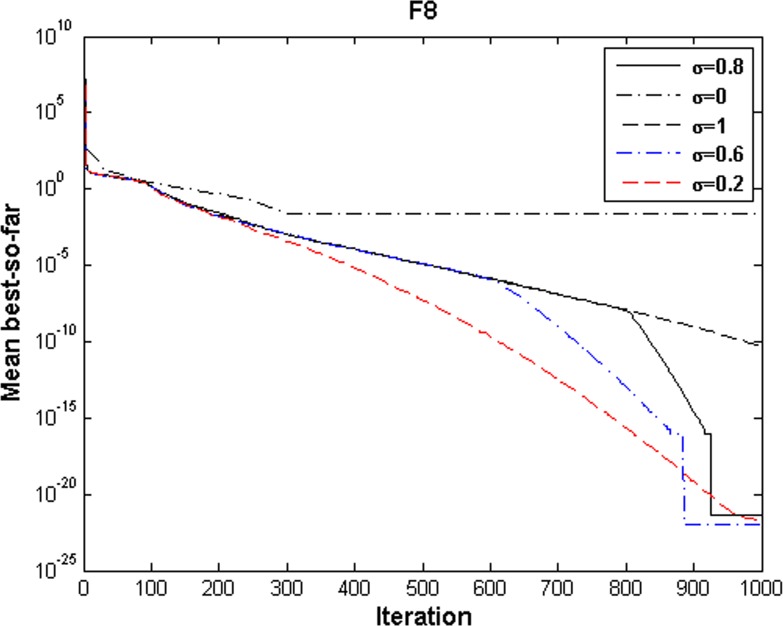
Performance comparison of different values of *σ* for multimodal function.

According to Fig 3, selecting the value of *σ* from 0.2 to 0.8 yields better results, although, the algorithm is less sensitive with respect to this parameter.

Effect of the parameter *σ* on optimization performance was studied on multimodal function F8 and the result is shown in Fig 3. In this function, the special case of ECGSA with only exploration ability (*σ* = 1) cannot achieve the best solution. The lower values of *σ* produce significantly better performance than *σ* = 1 consistently due to exploitative nature of ECGSA with *σ*≤0.8. In the multimodal case, the rapid convergence of *σ* = 0 is premature and the algorithm stagnates at local minima. ECGSA with σ = 1 lacks the exploitative ability almost completely, thus it is out performed by ECGSA with 0.2≤*σ*≤0.8. The interval 0.2≤*σ*≤0.8 produces a balanced exploration and exploitation ability as is obvious in F8. This case shows that there is negligible variation in performance in that interval, which indicates that the choice of σ is not critical. Only extreme values of *σ* should be avoided in real world optimization problems in order to maintain robustness of ECGSA.

The proposed dynamic gravitational damping coefficient somehow is similar to the temperature value of the SA algorithm [32] which controls the exploration and exploitation search abilities trade-off of the algorithm, but they are implemented via different equations.

## 4.Verification of the Proposed ECGSA

For verification, ECGSA is tested on eight representative standard benchmark functions along with six composite test functions. All the test functions are to be minimized and the relevant information can be found in [20] and [33] for the standard benchmark functions and the composite test functions, respectively.

For the proposed ECGSA, *N* is set to 50, *t*_*max*_ is set to1000, *G*_*0*_ is set to 100, αmin and *α*_*max*_ are set to 10 and 25, respectively, and *σ* is set to 0.6. The other parameters of ECGSA are set the same as in the standard GSA [20].

The standard benchmark functions are summarized in Tables 1 and 2 for unimodal and multimodal functions, respectively. The results of applying the algorithm to the unimodal and multimodal benchmark functions over 30 runs are reported in Tables 3 and 4, respectively. According to the tables, the consequences are reported for mean, median and best values of the obtained solutions, and the standard deviation value of them. Since GSA was shown to outperform similar algorithms like PSO and GA for the benchmark functions [20], the results are compared with GSA and some of its variants.

**Table 1:** Unimodal benchmark functions.

Test function	Search space (S)	Minimum value (*f*_*opt*_)
$F_{1}(X)=\sum_{i=1}^{n}x_{i}^{2}$	[-100,100]^n^	0
$F_{2}(X)=\sum_{i=1}^{n}\left|x_{i}\right|+\prod_{i=1}^{n}\left|x_{i}\right|$	[-10,10]^n^	0
$F_{3}(X)=\sum_{i=1}^{n}{\left(\sum_{j=1}^{i}x_{j}\right)}^{2}$	[-100,100]^n^	0
$F_{4}(X)=\text{max}\{\left|x_{i}\right|,1\leq i\leq n\}$	[-100,100]^n^	0
$F_{5}(X)=\sum_{i=1}^{n}\left[100{\left(x_{i+1}-x_{i}^{\;2}\right)}^{2}+{\left(x_{i}-1\right)}^{2}\right]$	[-30,30]^n^	0

**Table 2:** Multimodal benchmark functions.

Test function	Search space (S)	Minimum value (*f*_*opt*_)
$F_{1}(X)=\sum_{i=1}^{n}x_{i}^{2}$	[-100,100]^n^	0
$F_{2}(X)=\sum_{i=1}^{n}\left|x_{i}\right|+\prod_{i=1}^{n}\left|x_{i}\right|$	[-10,10]^n^	0
$F_{3}(X)=\sum_{i=1}^{n}{\left(\sum_{j=1}^{i}x_{j}\right)}^{2}$	[-100,100]^n^	0
$F_{4}(X)=\text{max}\{\left|x_{i}\right|,1\leq i\leq n\}$	[-100,100]^n^	0
$F_{5}(X)=\sum_{i=1}^{n}\left[100{\left(x_{i+1}-x_{i}^{\;2}\right)}^{2}+{\left(x_{i}-1\right)}^{2}\right]$	[-30,30]^n^	0

**Table 3:** Minimization result of the unimodal benchmark functions in Table 1.

Function	Method	Mean	Median	Best	Standard Deviation
F_1_	GSA	2.36×10^−17^	2.20×10^−17^	1.09×10^−17^	6.52×10^−18^
F_1_	MGSA[34]	1.18×10^−17^	1.16×10^−17^	7.66×10^−18^	2.95×10^−18^
F_1_	**ECGSA**	**1.06×10** ^**−20**^	**1.03×10** ^**−20**^	**4.32×10** ^**−21**^	**4.15×10** ^**−21**^
F_2_	GSA	2.44×10^−8^	2.41×10^−8^	1.87×10^−8^	3.22×10^−9^
F_2_	MGSA[34]	1.77×10^−8^	1.68×10^−8^	1.17×10^−8^	3.36×10^−9^
F_2_	**ECGSA**	**2.86×10** ^**−9**^	**2.06×10** ^**−9**^	**3.13×10** ^**−10**^	**2.56×10** ^**−9**^
F_3_	GSA	255.29	250.64	88.41	77.41
F_3_	MGSA[34]	9.49	8.28	0.13	9.04
F_3_	MGSA[35]	27.1	26.22	8.33	10.36
F_3_	SLGSA[28]	16.04	10.80	7.09	10.12
F_3_	**ECGSA**	**1.55×10** ^**−3**^	**1.35×10** ^**−3**^	**1.22×10** ^**−4**^	**0.0011**
F_4_	GSA	4.91×10^−9^	3.62×10^−9^	2.08×10^−9^	5.85×10^−9^
F_4_	OBGSA[36]	5.11×10^−9^	4.89×10^−9^	3.36×10^−9^	1.46×10^−9^
F_4_	SLGSA[28]	1.11×10^−9^	1.12×10^−9^	8.52×10^−9^	1.09×10^−9^
F_4_	**ECGSA**	**2.93×10** ^**−9**^	**2.97×10** ^**−9**^	**1.03×10** ^**−9**^	**1.12×10** ^**−9**^
F_5_	GSA	28.23	26.10	25.77	11.19
F_5_	MGSA[34]	24.60	24.34	23.54	1.004
F_5_	MGSA[35]	26.01	25.99	25.65	0.177
F_5_	SLGSA [28]	25.05	25.12	23.86	0.260
F_5_	**ECGSA**	**22.6**	**22.6**	**22.1**	**0.169**

**Table 4:** Minimization result of multimodal benchmark functions in Table 2.

Function	Method	Mean	Median	Best	Standard Deviation
F_6_	GSA	-2.69×10^3^	-2.66×10^3^	-3.66×10^3^	385.822
F_6_	MGSA[34]	-6.81×10^3^	-6.94×10^3^	-8.66×10^3^	1.18×10^3^
F_6_	**ECGSA**	**-7.85×10** ^**3**^	**-7.77×10** ^**3**^	**-8.98×10** ^**3**^	**229.995**
F_7_	GSA	4.40	4.29	1.38	1.72
F_7_	MGSA[34]	1.61	1.95	0.17	1.38
F_7_	**ECGSA**	**2.48×10** ^**−2**^	**1.48×10** ^**−2**^	**0.00×10** ^**0**^	**0.027**
F_8_	GSA	5.68×10^−2^	8.42×10^−20^	8.42×10^−20^	0.109
F_8_	MGSA[34]	0.02	1.10×10^−19^	7.32×10^−20^	0.05
F_8_	OBGSA[36]	0.0017	6.24×10^−4^	2.76×10^−5^	0.003
F_8_	SLGSA [28]	5.69×10^−19^	5.72×10^−19^	2.72×10^−19^	1.65×10^−19^
F_8_	**ECGSA**	**1.02×10** ^**−22**^	**8.68×10** ^**−23**^	**2.82×10** ^**−23**^	**7.14×10** ^**−23**^

As it can be seen, ECGSA outperforms GSA and its variants for all the benchmark functions tested in this study. The standard deviation of the optimization result from different runs also verifies the robustness of the proposed algorithm.

According to Table 3, the proposed ECGSA can optimize unimodal functions more consistently than the GSA variants. This shows that the exploitation search ability of ECGSA is significantly better than GSA and its variants as the unimodal functions require strong exploitation ability for optimization because of the convexity of such problems.

**Fig 4:**
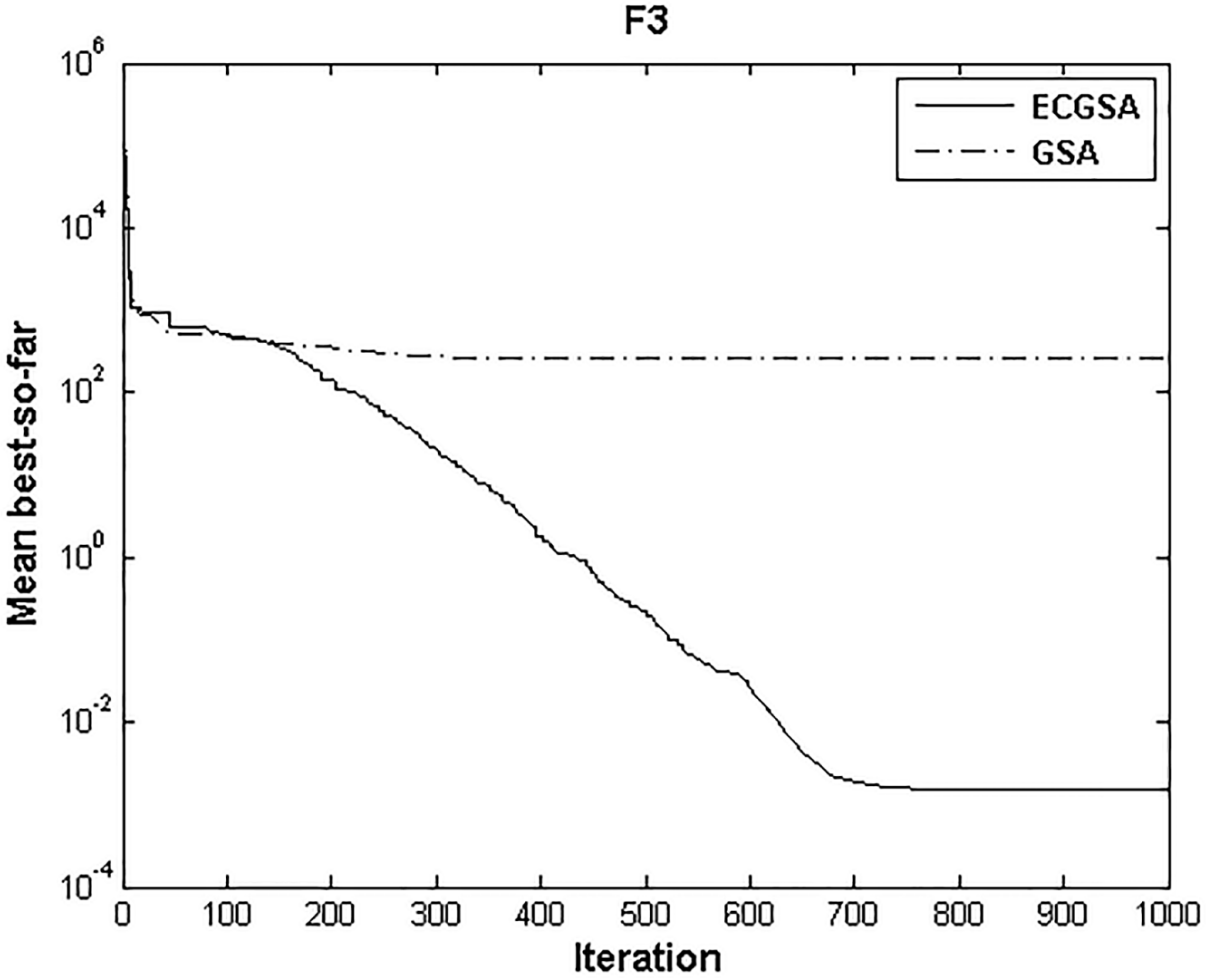
Performance comparison of ECGSA and GSA for F_3._

The convergence characteristic of the proposed algorithm with respect to the standard GSA is shown on Fig 4 for validating the results related to the unimodal functions. The premature convergence of GSA for F_3_ occurs at about 200 to 300 iterations. But due to setting a low value for *α*, ECGSA can resist premature convergence. Considering the best fitness function evaluations for improving the optimal search trajectory results in good exploration. This strategy would result in poor convergence speed if *α*_*min*_ is used throughout the optimization process. At 600 iterations, ECGSA optimization of F_3_ shows a sharp decrease due the start of the exploitation phase (*α* increasing linearly). The results suggest that a good trade-off has been established in the exploitation and exploration aspects of ECGSA for unimodal functions.

Table 4 presents the results of optimization of multimodal functions in Table 2 using ECGSA, GSA and its variants. The performance of ECGSA is significantly better than GSA and its variants as illustrated by the mean optimization results in Table 4. Furthermore, ECGSA also has lower standard deviation showing that it is more stable than GSA and its variants. This establishes the superior ability to escape local minima and exploration performance of ECGSA.

**Fig 5:**
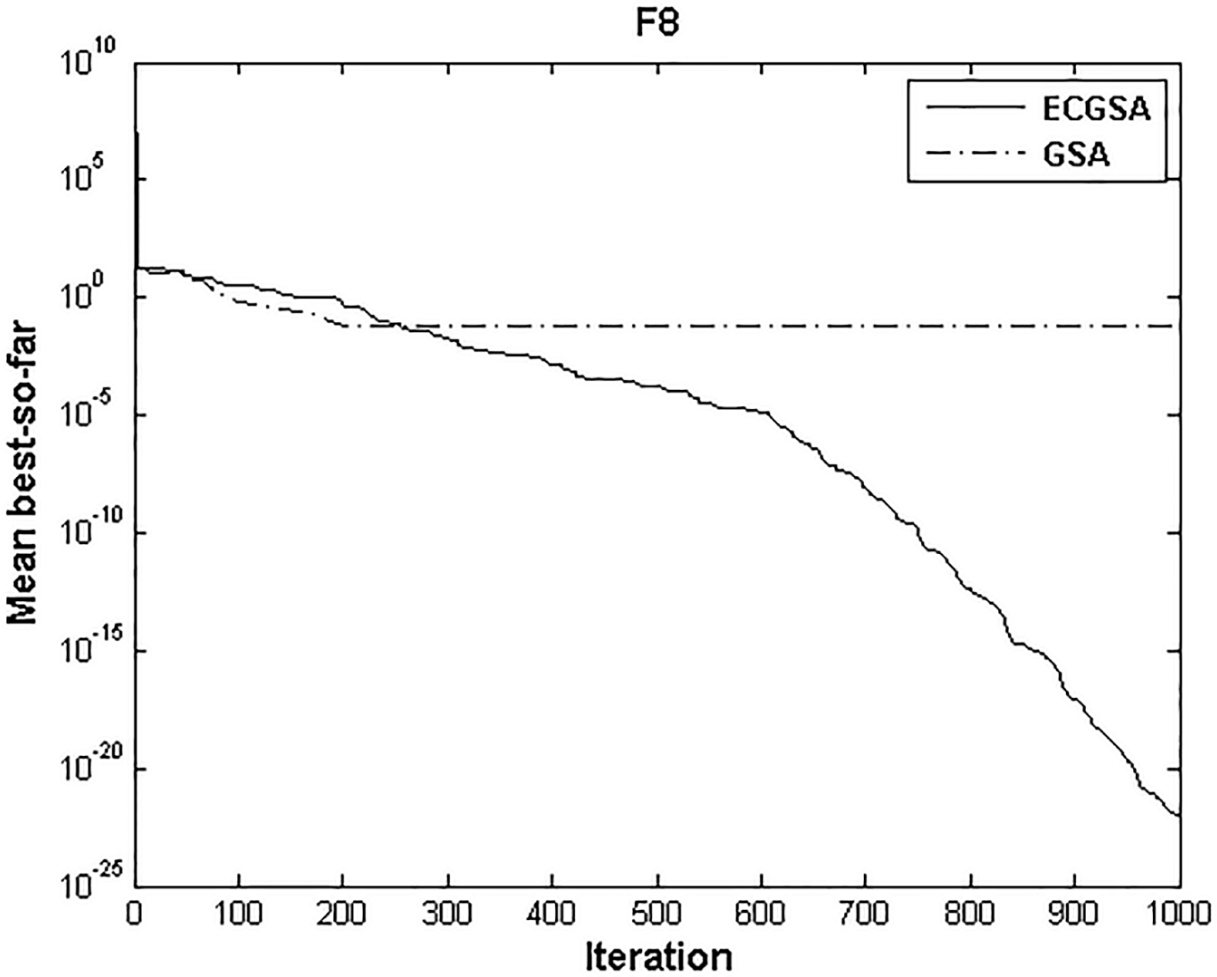
Performance comparison of ECGSA and GSA for F_8._

Fig 5 shows similar traits to Fig 4. The exploration phase of ECGSA is benefitted by lower value of *α*_*min*_ compared to *α* of GSA. Thus GSA prematurely converges to suboptimal local minima before 200 iterations. During the exploitation phase, once *α* begins to increase, the sharp convergence leads to a rapid drop after 600 iterations in Fig 5. Using the best found experiments of agents as their present positions results in the ECGSA curve falling away from the GSA curve in both Figs 4 and 5, even before the *α* starts to vary dynamically. This clearly indicates that using the best found fitness function evaluations of agents as their present positions results in improved exploration in the early phase. As *α* starts to vary at 600th iteration, the best fitness function evaluations of agents improved exploration allows greater exploitation by attracting the agents towards their best locations, rather than the current, possibly suboptimal, location. Thus, the convergence process is more stable, allowing the proposed algorithm to optimize multiple complex composite test functions more accurately and efficiently than most well-known heuristic algorithms.

The Wilcoxon’s Rank Sum test [37] is also used to analyze and to validate the proposed algorithm. Since the underlying probability density function for the algorithm solutions is unknown, techniques like T-test are unusable. Table 5 illustrates the Wilcoxon's rank-sum test between GSA and ECGSA for the benchmark functions. Sufficiently large sample sizes are considered to ensure a viable comparison.

**Table 5:** Wilcoxon's rank-sum test between GSA and ECGSA for benchmark functions.

Function	N1	N2	Sum	Expectation	Std. Error	State	P-Value
F1	30	30	1365	915	42.77	10.51	3.01×10^−11^
F2	30	30	1365	915	42.77	10.51	3.00×10^−11^
F3	30	30	1365	915	42.77	10.51	3.01×10^−11^
F4	30	30	1107	915	42.77	4.48	3.59×10^−6^
F5	30	30	1365	915	42.77	10.51	3.01×10^−11^
F6	30	30	1365	915	42.77	10.51	2.70×10^−11^
F7	30	30	1365	915	42.77	10.51	1.94×10^−11^
F8	30	30	1361	915	42.77	10.42	3.00×10^−11^

The table shows extremely low p-values for the results indicating that ECGSA can be considered superior to GSA for both unimodal and multimodal benchmark functions within a confidence interval of 99.9% (p-value<0.001).

For more verification, we have tested ECGSA on the composite benchmark functions introduced recently to validate the performance of heuristic algorithms [33]. These functions combine multiple features of various unimodal and multimodal functions. These functions test several properties of the proposed algorithm simultaneously. The results of the algorithm for the composite function optimizations are from 20 runs, for the sake of comparison, as suggested in literature [33].

Table 6 compares the performance of the proposed algorithm versus several well-known heuristic methods. The results clearly indicate that the proposed ECGSA achieves significantly better solutions than all other algorithms considered in Table 6. Only DE marginally outperforms GSA in CF6. However, ECGSA comprehensively outperforms DE for the other 5 composite test functions. ECGSA shows high stability as it exhibits the lowest standard deviation for most of the composite test functions.

**Table 6:** Composition tests functions.

Function		PSO [33]	CPSO [33]	CLPSO [33]	CMA-ES [33]	G3-PCX [33]	DE [33]	GSA	ECGSA
CF1	Mean	1.00×10^2^	1.56×10^2^	5.73×10^−8^	1.00×10^2^	6.00×10^1^	6.74×10^−2^	5×10^0^	**2.19×10** ^**−21**^
CF1	Std	8.16×10^1^	1.34×10^2^	1.03×10^−7^	1.88×10^2^	6.99×10^1^	1.10×10^−1^	2.23×10^1^	**1.04×10** ^**−21**^
CF2	Mean	1.55×10^2^	2.42×10^2^	1.91×10^1^	1.16×10^2^	9.26×10^1^	2.87×10^1^	1.90×10^2^	**5.12×10** ^**0**^
CF2	Std	1.31×10^2^	1.48×10^2^	1.47×10^1^	1.51×10^2^	9.90×10^1^	8.62×10^0^	5.52×10^1^	**1.13×10** ^**1**^
CF3	Mean	1.72×10^2^	3.62×10^2^	1.32×10^2^	2.14×10^2^	3.19×10^2^	1.44×10^2^	1.13×10^2^	**1.09×10** ^**2**^
CF3	Std	3.28×10^1^	1.96×10^2^	2.00×10^1^	7.41×10^1^	1.25×10^2^	1.94×10^1^	7.57×10^1^	**9.33×10** ^**1**^
CF4	Mean	3.14×10^2^	5.22×10^2^	3.22×10^2^	6.16×10^2^	4.92×10^2^	3.24×10^2^	4.27×10^2^	**2.55×10** ^**2**^
CF4	Std	2.00×10^1^	1.22×10^2^	2.74×10^1^	6.71×10^2^	1.42×10^2^	1.47×10^1^	1.67×10^2^	**3.79×10** ^**1**^
CF5	Mean	8.34×10^1^	2.55×10^2^	5.37×10^0^	3.58×10^2^	2.60×10^1^	1.07×10^1^	2.33×10^2^	**0.72×10** ^**0**^
CF5	Std	1.01×10^2^	1.75×10^2^	2.60×10^0^	1.68×10^2^	4.15×10^1^	2.60×10^0^	6.18×10^1^	**1.00×10** ^**0**^
CF6	Mean	8.61×10^2^	8.53×10^2^	5.01×10^2^	9.00×10^2^	7.72×10^2^	4.90×10^2^	7.96×10^2^	**5.00×10** ^**2**^
CF6	Std	1.25×10^2^	1.27×10^2^	7.78×10^−1^	8.31×10^2^	1.89×10^2^	3.94×10^1^	1.00×10^2^	**0.20×10** ^**0**^

Table 7 confirms the superiority of proposed ECGSA over GSA using the Wilcoxon Rank Sum test. The p-values indicate that ECGSA achieves superior results than GSA within a confidence interval of 99.9% for all of the composite functions except CF3.

**Table 7:** Wilcoxon's rank-sum test between GSA and ECGSA for composition functions.

Function	N1	N2	Sum	Expectation	Std. Error	State	P-Value
CF1	20	20	610	410	23.38	8.55	6.77×10^−8^
CF2	20	20	512	410	23.38	4.36	3.23×10^−8^
CF3	20	20	418	410	23.38	0.34	0.36
CF4	20	20	488	410	23.38	3.33	4.24×10^−4^
CF5	20	20	602	410	23.38	8.21	1.65×10^−7^
CF6	20	20	610	410	23.38	8.55	2.43×10^−8^

## 5.MVDR Beamforming

MVDR is a minimum output energy (MOE) system based beamforming technique, as explicitly described previously [28]. MVDR keeps a distortionless main lobe response towards the wanted signal while simultaneously minimizing the array output power. The weights of MVDR can be written as [18]:

$$W_{MVDR}=\frac{R^{-1}a(\theta )}{a^{H}(\theta )R^{-1}a(\theta )}$$ (14)

where *R* is the covariance matrix, *H* is the Hermitation transpose and *a(θ)* is the steering vector of the desired signal. Where the steering vector *a(θ)* is given by:

$$a(\theta )=\left[\begin{array}{l} 1\\ \text{exp}\left\{j\frac{2\pi }{\lambda }(\text{sin}\;\theta_{i})d\right\}\\ \text{exp}\left\{j\frac{2\pi }{\lambda }(\text{sin}\;\theta_{i})(m-1)d\right\} \end{array}\right]$$ (15)

where *d* is the space between the elements of the antenna, *θ*_*i*_ is the desired angle, and *m* is the number of elements. However, typically, low nulling levels towards multiple interference sources is one of the major drawbacks of MVDR beamforming technique [3, 18, 38]. Therefore, many studies have tried to achieve nulls in the interference directions with various solutions [18, 38–39].

## 6.Model Application

The robustness and efficiency of the proposed method have been evaluated through eight benchmark functions and six composite test functions in the previous section. In this section, the calculation of signal to interference noise ratio (*SINR*) and applicability of the proposed method to increase *SINR* of MVDR technique is presented and it is compared with conventional MVDR, MVDR-PSO and MVDR-GSA. Two cases of interference scenarios are considered in this study. Both cases have one user at 0°. The first case contains a single interference source positioned at 30° and the second case considers two interference sources at 30° and 50°. The employment of the ECGSA for MVDR analysis has been carried out using MATLAB®.

### 6.1 Signal to interference and noise ratio calculation

In this paper, the ECGSA was employed to enhance the MVDR beamforming technique performance by increasing the *SINR* value of four element array. The MVDR integrated with ECGSA, GSA and PSO method will optimize the *SINR* via the complex weights, as described in detail previously [28].

In these algorithms, the *w*_*mvdr*_ (MVDR weight vector) will be retained for one member in the initial population, whereas, the rest of the population will be initialized randomly as shown in (17). This system will initiate by generating the *N* agents, which is indicated by *W*_*N*_ weight vectors. The weight vectors in every agent contain *M* number of weight vectors, where *M* is the number of elements in the array.

$$W_{NM}=x_{i}^{d}$$ (16)

where population size, *i*, in ECGSA, GSA and PSO is set to *N*, and dimension *d* is equal to number of sensor *M* as shown in (16).

The weight vectors of the entire population can be illustrated in the matrix format below:

$$W_{NM}=\left[\begin{array}{llll} w_{mvdr1} & \;w_{mvdr2} & \;w_{mvdr3} & \;w_{mvdr4}\\ w_{21} & \;w_{22} & \;w_{23} & \;w_{24}\\ w_{31} & \;w_{32} & \;w_{33} & \;w_{34}\\ . & \;. & \;. & \;.\\ . & \;. & \;. & \;.\\ w_{n1} & \;w_{n2} & \;w_{n3} & \;w_{n4} \end{array}\right]$$ (17)

Where

$$W_{NM}=\;\text{Weight vectors of N particles with M sensors in each antenna}$$

$$W_{mvdr}=\;\text{weight vectors from conventional MVDR beamformer}$$

**Fig 6:**
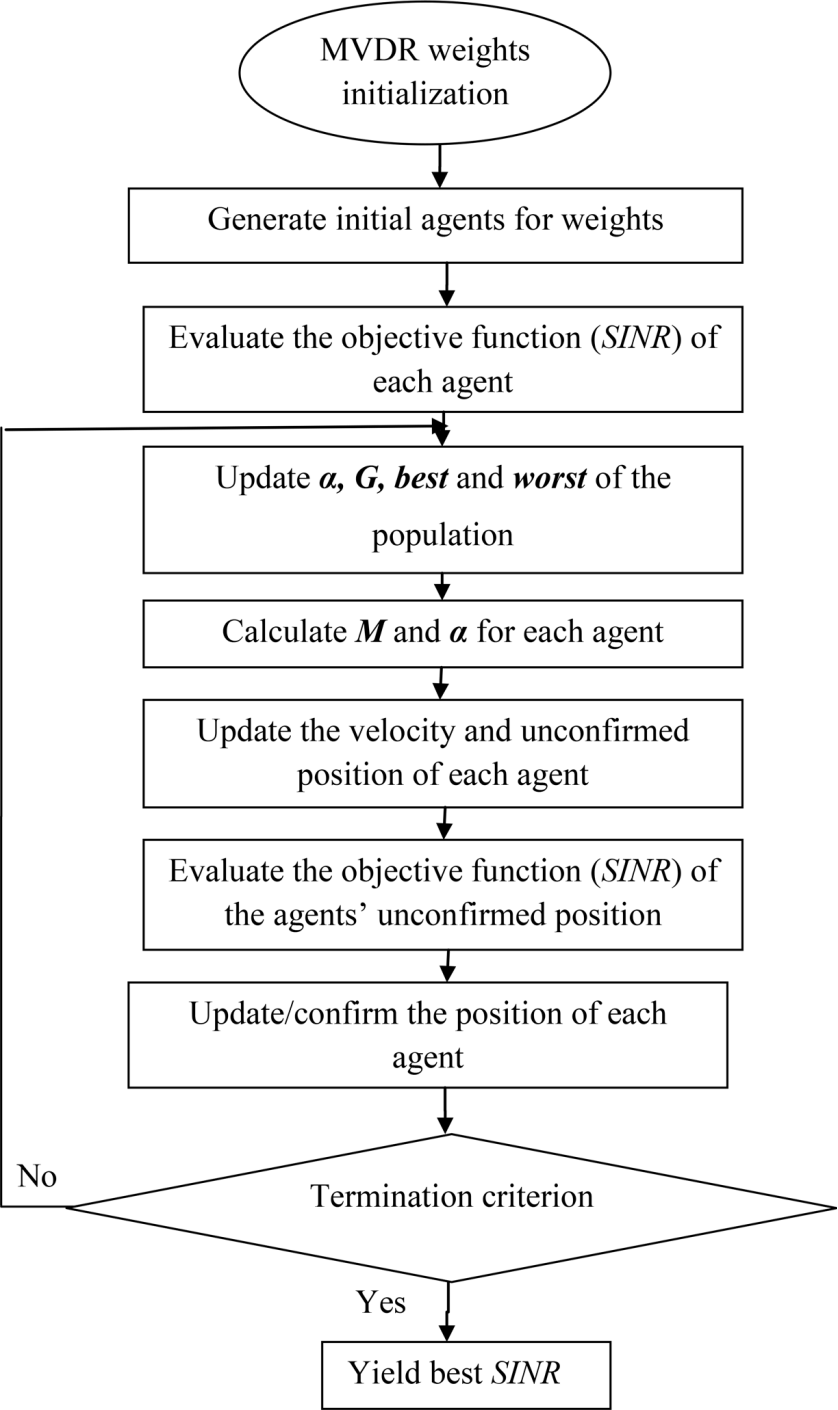
The simplified flowchart of ECGSA beamforming.

The fitness function, according to literature [28], is the *SINR*, as shown in (18). Thus, the optimization processes will try to maximize the fitness function and find the corresponding optimal weight vectors as shown in Fig 6.


$$FitnessFunction(F_{N})=\frac{P_{U}}{\sum_{i=1}^{I}P_{i}+N}$$ (18)

Where

$$P_{u}=\text{Power of Target user}$$

$$P_{i}=\text{Power of Interference at}\;i\;\text{interference}$$

I=Number of Interference sources

N=Noise

### 6.2 Simulation

In this section, multiple interference scenarios were simulated to validate the proposed approach for real world applications. The parameters of GSA are chosen according to the guidelines and recommendations presented in [20]. These configurations of GSA have also been utilized extensively after the development of GSA [34–36]. In all of the PSO-based simulations, the inertia weight *w* = 0.7298, and the acceleration constants *c*_*1*_ = *c*_*2*_ = 1.49618 were utilized. These values are chosen based on the common settings in the literature [40–42]. The algorithms are simulated 20 times with maximum number of iteration set to 100, and the best results are recorded.

### 6.2.1 Case 1: One user one interferences

**Fig 7:**
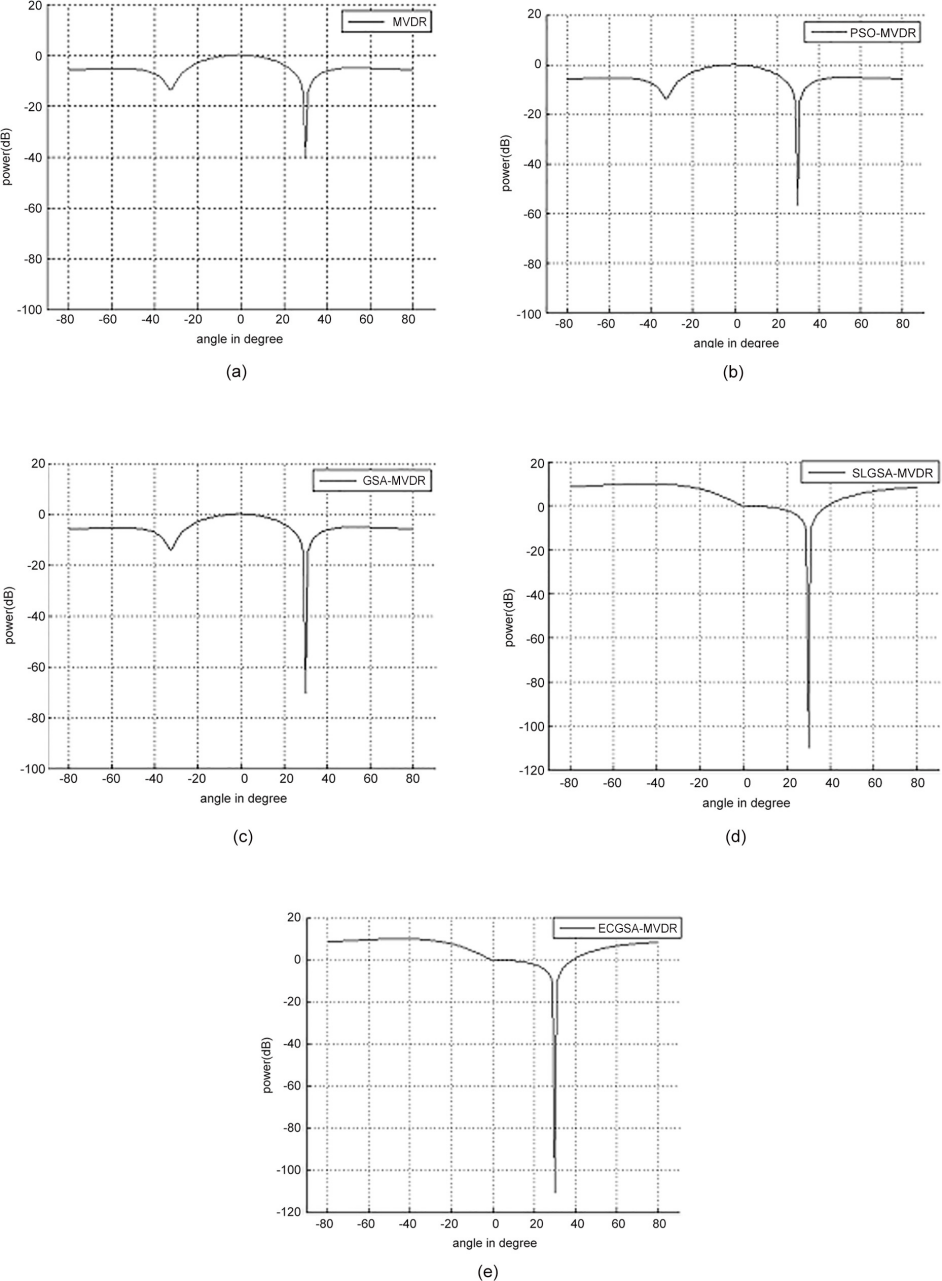
Comparison of performance of power response with 100 iterations for user at 0° with interference at 30°. (a) MVDR, (b) PSO-MVDR, (c) GSA-MVDR, (d) SLGSA-MVDR [28] and (e) ECGSA-MVDR.

One interference source at 30° and user at 0° has been assumed in the first case study. Table 8 illustrates the weights corresponding to optimal solutions determined by conventional MVDR, PSO-MVDR, GSA-MVDR, SLGSA-MVDR [28] and ECGSA-MVDR. These weights yield the power response as shown in Fig 7.

**Table 8:** Comparison of weight vectors for conventional MVDR, PSO-MVDR, GSA-MVDR, SLGSA-MVDR [28] and ECGSA-MVDR for user at 0° and interferences at 30°.

Name	MVDR	PSO-MVDR	GSA-MVDR	SLGSA-MVDR	ECGSA-MVDR
Weights	0.2986 + 0.0122i	0.2990 + 0.0121i	0.3000+ 0.0103i	0.170 +2.4159i	0.1708 + 2.4159i
	0.1967 + 0.0212i	0.1964 +0.0210i	0.1976 + 0.0196i	3.3166–2.2630i	3.3166–2.2630i
	0.3367–0.0166i	0.3366–0.0164i	0.3369–0.0177i	-2.7788–0.6312i	-2.7788–0.6312i
	0.1678–0.0168i	0.1679–0.0165i	0.1694–0.0172i	0.2694 + 0.6866i	0.2694 + 0.6866i

Table 9 illustrates improvement of *SINR* by PSO and GSA, compared to conventional MVDR, are 38.89% and 65.04%, respectively. The SLGSA [28] and ECGSA show the best performance of 72.17% percent improvement. The *SINR* of 69.99 is the global maximum for this case, which is achieved by both SLGSA-MVDR and ECGSA-MVDR as only one deep null is required. Failure of MVDR, GSA-MVDR and PSO-MVDR to achieve sufficiently deep nulls clearly illustrates their limitations.

**Table 9:** Comparison of *SINR* calculation for conventional MVDR, PSO-MVDR, GSA-MVDR, SLGSA-MVDR [28] and ECGSA-MVDR for user at 0° and interference at 30°.

Name	SINR(dB)
MVDR	40.65
PSO-MVDR	56.47
GSA-MVDR	67.10
SLGSA-MVDR	69.99
ECGSA-MVDR	69.99

### 6.2.2 Case 2: One user two interferences

**Fig 8:**
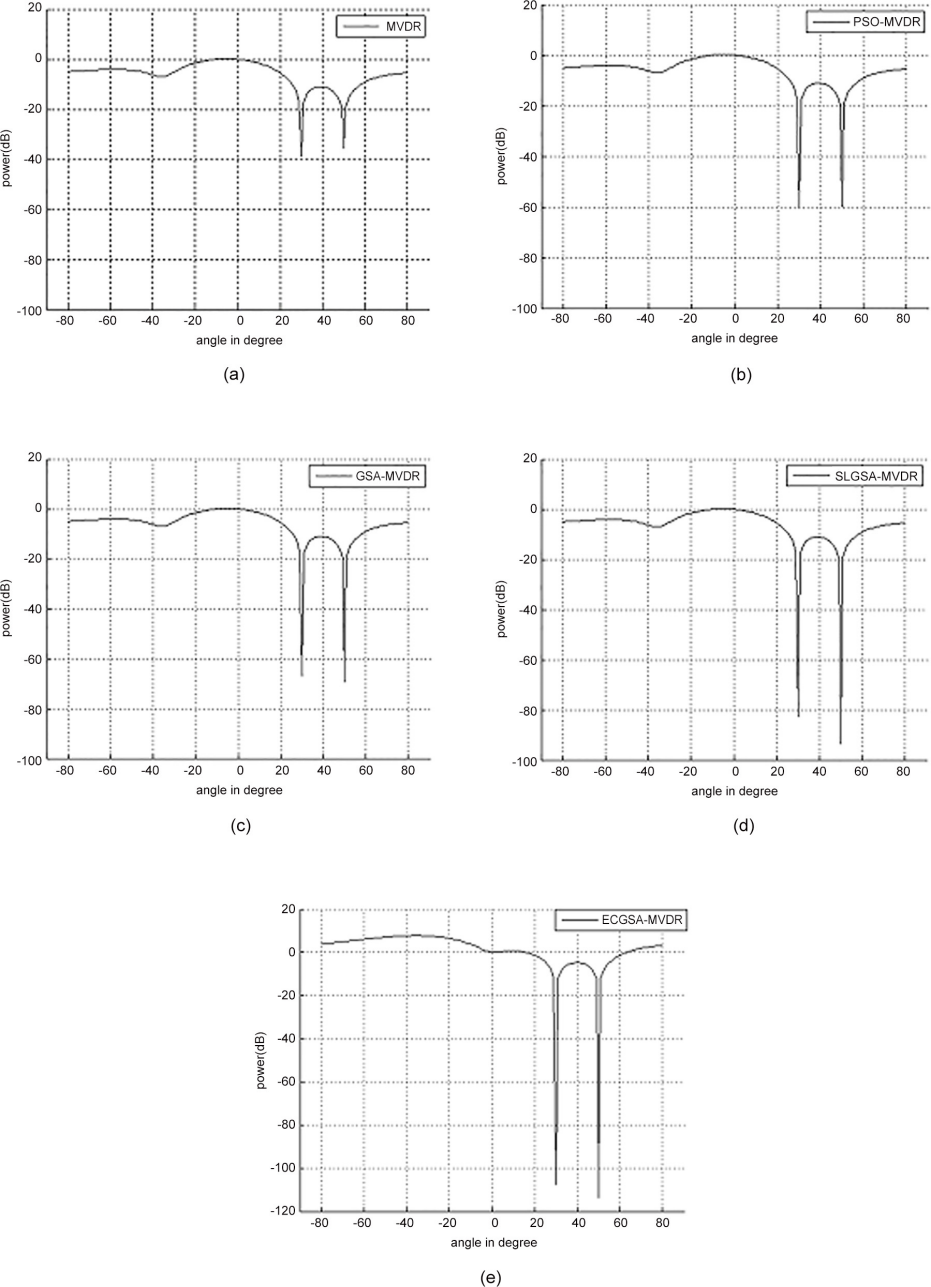
Comparison of performance of power response with 100 iterations for user at 0°with two interferences at 30° and 50°. (a) MVDR, (b) PSO-MVDR, (c) GSA-MVDR, (d) SLGSA-MVDR [28] and (e) ECGAS-MVDR.

Two interferences at 30°, 50° and user at 0°has been assumed as the second case study. The weights corresponding to optimal solutions determined by the different beamforming algorithms are shown in Table 10. The resulting power responses from these weights are illustrates in Fig 8.

**Table 10:** Comparison of weight vectors for conventional MVDR, PSO-MVDR, GSA-MVDR, SLGSA-MVDR [28] and ECGSA-MVDR for user at 0° and interferences at 30°and 50°.

Name	MVDR	PSO-MVDR	GSA-MVDR	SLGSA-MVDR	ECGSA-MVDR
Weights	0.2233 + 0.1492i	0.2233 + 0.1491i	0.2234 + 0.1491i	0.2231 +0.1491i	0.9211–0.0153i
	0.3453–0.0650i	0.3453–0.0654i	0.3453–0.0651i	0.3454 0.0650i	-0.6394–1.4880i
	0.2387–0.003i	0.2384–0.0034i	0.2389–0.0030i	0.2387 0.0035i	-1.4874 + 1.5400i
	0.1925–0.0806i	0.1927–0.0805i	0.1931–0.0807i	0.1926 0.0807i	0.9159 + 0.9205i

Table 11 shows that the improvement of *SINR* by PSO, GSA and SLGSA [28], compared to conventional MVDR method, are 67.75%, 87.84% and 105.84%, respectively. It can be seen that ECGSA shows the best performance, which is 106.58% percent of improvement. The increase in number of interference sources inherently increases the difficulty of the optimization problem. The superiority of ECGSA-MVDR in case 2 is more apparent than in case 1, especially for SLGSA-MVDR [28]. Thus, ECGSA is more versatile and robust than GSA, PSO and SLGSA.

**Table 11:** Comparison of *SINR* calculation for conventional MVDR, PSO-MVDR, GSA-MVDR, SLGSA-MVDR [28] and ECGSA-MVDR for user at 0° and interference at 30°and 50°.

Name	SINR(dB)
MVDR	33.88
PSO-MVDR	56.85
GSA-MVDR	63.65
SLGSA-MVDR	69.74
ECGSA-MVDR	69.99

Overall, the performance disparity between proposed algorithm and SLGSA is expected to increase further with higher number of interference sources. In case 1, as SLGSA-MVDR is able to locate the optimal solution for one interference source, there is no potential for further improvement. As a result, the proposed ECGSA algorithm provides the same result. Subsequently, case 2 was investigated to reveal the minor, yet tangible, SINR improvement by the proposed algorithm over SLGSA-MVDR. The superior performance of ECGSA in case 2, along with the results of the benchmark problems, establishes ECGSA as a suitable interference mitigation algorithm.

## 7.Conclusions

An experience oriented-convergence improved gravitational search algorithm with dynamic gravitational damping coefficient, α, and searching through the best experiments is presented in this paper. GSA outperforms most known heuristic algorithms but the constant α and unstable search trajectory without any feedback to their best experiments decrease the effectiveness of GSA. Consequently, the proposed dynamic gravitational damping coefficient alters the gravitational constant, resulting in better balance between exploration and exploitation. Using the best found experiments of agents as their present positions provides steadier trajectory, as GSA uses the stochastically varying current position. This modified system exhibits better convergence rate, precision, stability and robustness compared to GSA on fourteen unimodal, multimodal and composite test functions. The proposed algorithm has been applied on MVDR beamforming problems in order to verify the effectiveness of ECGSA in real world applications. Results show superior performance of ECGSA-MVDR in comparison to the conventional MVDR, MVDR-PSO, MVDR-GSA and MVDR-SLGSA due to its ability in achieving higher *SINR*.

## Supporting Information

S1 File Data for ECGSA result in Tables 3 and 4. (XLSX)

S2 File Data for ECGSA and GSA results in Table 6. (XLSX)
